# Pb Removal Efficiency by Calcium Carbonates: Biogenic versus Abiogenic Materials

**DOI:** 10.1021/acs.cgd.3c00517

**Published:** 2023-12-05

**Authors:** Ana Roza-Llera, Fulvio Di Lorenzo, Sergey V. Churakov, Amalia Jiménez, Lurdes Fernández-Díaz

**Affiliations:** † Department of Geology, University of Oviedo, Oviedo 33005, Spain; ‡ Laboratory for Waste Management, Paul Scherrer Institute, Villigen 5232, Switzerland; § Laboratory for Waste Management, Paul Scherrer Institute, Villigen 5232, Switzerland; ∥ Department of Geology, University of Oviedo, Oviedo 33005, Spain; ⊥ Department of Mineralogy and Petrology, Complutense University of Madrid, Madrid 28040, Spain

## Abstract

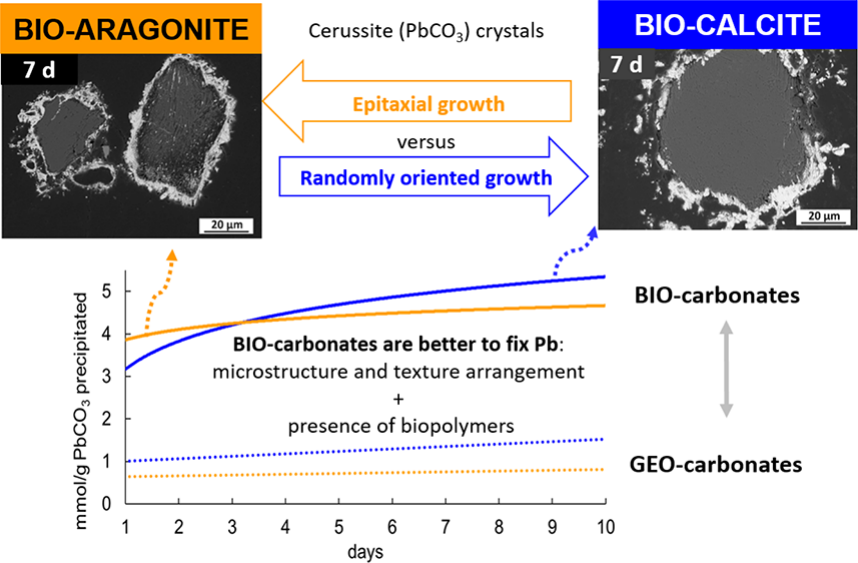

The sorption of heavy metals on mineral surfaces plays a key role in controlling the fate and bioavailability of harmful elements through dissolution–precipitation reactions. Here, we investigate the efficiency of Pb removal from highly contaminated waters by two calcium carbonate hard tissues, scallop shells (up to 99.9 mol %; -biocalcite) and cuttlefish bones (up to 90.0 mol %; bioaragonite), which template the precipitation of the highly insoluble mineral cerussite (PbCO_3_). The experiments show that both biomaterials are about five times more effective Pb scavengers (5 mmol of cerussite precipitated/g sample) than their inorganic counterparts (∼1 mmol/g). We relate this enhanced Pb scavenging capacity of biocarbonates to their composite organic–inorganic nature, which modulates their specific nano- and microstructural features and defines their larger surface areas, solubility, and reactivity compared to those of their inorganic counterparts. The oriented growth of cerussite progressively passivates the bioaragonite surface, reducing its long-term Pb scavenging capacity. In contrast, the randomly oriented growth of cerussite crystals on biocalcite prevents surface passivation and explains why biocalcite outperforms bioaragonite as a long-term Pb scavenger. The use of biocarbonates could be a key for designing more efficient decontamination strategies for heavy metal-polluted waters.

## Abstract 2

This work studies the ability of two calcium biocarbonate skeletons to scavenge dissolved Pb and effectively immobilize this contaminant within the crystalline structure of poorly soluble cerussite (PbCO_3_) by a surface dissolution−precipitation reaction. It is shown that the enhanced Pb scavenging capacity of biocarbonates is attributed to their specific microstructure that grants them significantly larger specific areas and reactivity compared to their inorganic counterparts. More efficient Pb sequestration by biocalcite as compared to bioaragonite is explained by differences in the degree of structural matching between the substrate and the precipitate.

## Introduction

The volume of soils and groundwater contaminated by heavy metals due to industrial activities such as pharmacy, nuclear, chemical, and battery manufacturing steadily has increased over the past century.^1^ Moreover, water pollution by heavy metals in abandoned mines and accidental spillages has generated important environmental damages and threatened drinking water supplies in numerous countries around the world.^2,3^ In recent years, a variety of methods such as coagulation and flocculation, electrocoagulation, electrolysis, and electro-deionization has been applied to decontaminating heavy metal-polluted waters.^4−7^ Most of these methods are efficient, but they also are very costly in energy, which makes them unfeasible for the economies of those countries with the most important problems of heavy metal contamination. In this scenario, the design of cheap, efficient strategies for decontaminating heavy metal-polluted water is essential.^8−11^

Among heavy metals, lead is one of the most hazardous for the environment and poses the most serious risks to human health as it can damage the circulatory, nervous, endocrine, and immune systems of the human body.^2,12^ Furthermore, due to its mutagenic, teratogenic, and carcinogenic potential, Pb is especially dangerous for vulnerable groups, such as children, pregnant women, and the elderly.^1,3,13−18^ It has been demonstrated that the mobility of Pb in the environment can be controlled by its interactions with mineral surfaces through different sorption mechanisms (absorption, adsorption, and surface precipitation).^19−31^ Different authors have demonstrated that the interaction between Pb-polluted waters and calcium carbonate polymorphs calcite and aragonite leads to the precipitation of Pb–carbonate and Pb removal from the liquid phase.^21,28−30,32−34^ Most of these studies have been conducted using calcite and aragonite samples of abiogenic origin.^25,35−39^ Despite obtaining very promising results, few studies have been focused on exploring the potential of biogenic CaCO_3_ materials (BIO-CaCO_3_ hereafter) as a heavy metal scavenger.^33,40,41^ Recent studies show that BIO-CaCO_3_, such as marine shells, eggshells, sepia cuttlebone, etc., widely outperform inorganic CaCO_3_ taking up heavy metals from polluted waters with triple efficiency.^33,42,43^ The fact that main waste products of the mariculture industry like shells of bivalves or cuttlebones of cephalopods are BIO-CaCO_3_ of problematic disposal strengthens the interest of using these materials as toxic elements scavengers. Up to now, only a small fraction of these wastes are being recycled in the production of fertilizers and as animal food additives.^44^ It is therefore worth exploring the heavy metal decontamination strategies based on the use of readily available BIO-CaCO_3_.

Most biogenic calcium carbonates are hierarchically structured composite materials that comprise two intimately interlinked components: pliant polymers (up to 10 wt %) and hard, brittle minerals (≥90 wt %).^45^ The mineral component mainly consists of nanoparticulate calcite and/or aragonite, which appear arranged in mineral units with definite textures and microstructures.^46−53^ The biopolymers are complex assemblies of polysaccharides, proteins, glycoproteins, and glycosaminoglycans, which form a network of fibrils surrounding BIO-CaCO_3_ mineral units. The texture and microstructure of biominerals as well as the composition and distribution of its biopolymers are species-specific and can even vary between different parts of the hard tissue.^48,54^

In this work, we study the uptake of Pb^2+^ by the surface of two highly BIO-CaCO_3_ materials, the shell of the bivalve *Chlamys opercularis* (*Aequipecten opercularis*), which is composed of calcite (BIO-CAL), and the cuttlebone of the cephalopod *Sepia officinalis*, which is composed of aragonite (BIO-ARG). Both species are popular seafoods whose hard tissues constitute important waste products from fishery, aquaculture, and canning industries. *C. opercularis* (*A. opercularis*) is an important fishery in North-Atlantic European countries, including UK, Ireland, France, Norway, and Spain, where annual landings are well over 30,000 tons.^55^ Similarly, *S. officinalis* is among the commercially most important species of cephalopod, constituting an appreciated fishery resource in Northeast Atlantic and Mediterranean waters.^56,57^ Total annual landings of *S. officinalis* in the English Channel between 2015 and 2020 ranged from 8.9 to 12.6 thousand metric tons.^58^

Aiming to evaluate the efficiency of Pb uptake by BIO-CaCO_3_ materials, we conducted batch experiments in which micrometer-sized fragments of the bivalve shell and the cephalopod cuttlebone were interacting with an acidic solution containing Pb. The X-ray diffraction and scanning electron microscopy analysis of the interacted samples allowed characterization of the nature and distribution of newly formed phases. In situ atomic force microscopy (AFM) observations of the BIO-ARG surface nanotopograhy in contact with water and a Pb-bearing aqueous solution provided information about the surface evolution in the course of Pb-carbonate precipitation. The results of Pb^2+^ uptake by BIO-CAL and BIO-ARG reported herein are compared to previously published data on Pb^2+^ sorption on the surface of geologic calcite and aragonite crystals. Differences in the Pb^2+^ scavenging capacity of BIO-CAL, BIO-ARG, and abiogenic carbonate minerals are interpreted on the basis of structural considerations, thermodynamic solubility, surface reactivity, and dissolution kinetics. The conclusions derived from this work provide clues that might help to optimize the use of BIO-CaCO_3_ material for remediation of water contaminated with heavy metals within the framework of a circular economy that promotes the recycling of waste materials, allowing for a reduction in the extraction of natural resources.

## Experimental Section

### Materials

**Figure 1:**
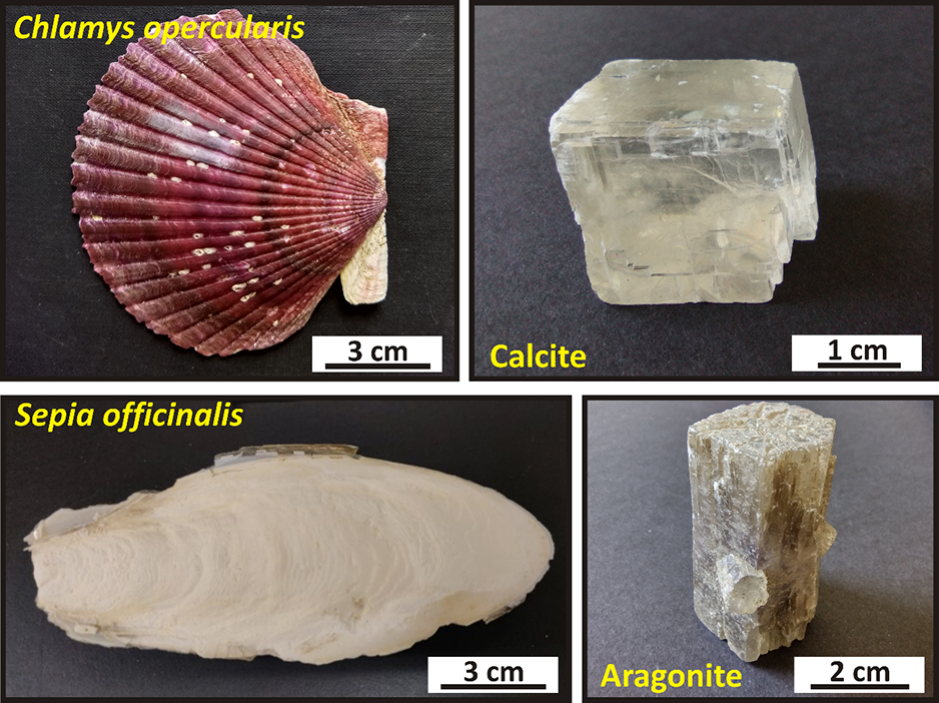
Overview images of biominerals used for the interaction experiments and the inorganics minerals used to compare the lead removal efficiency of Pb-bearing aqueous solutions. Images were taken prior to grinding.

Two different calcium carbonate hard tissues composed of calcite (BIO-CAL) and aragonite (BIO-ARG) were selected for this study: the calcitic shell of the scallop *C. opercularis* (*A. opercularis*) and the aragonitic cuttlebone of the cephalopod *S. officinalis*. The microstructure of these biogenic materials is quite different: like other bivalves, the shell of scallop consists of three superposed layers built up of long, tabular, lath-like calcite crystals defining a foliated microstructure.^48,59,60^ Mineral units in the shell *C. opercularis* (*A. opercularis*) are encased by very thin (20–50 nm) organic membranes and occlude finer fibril networks of biopolymers.^60^*Sepia* cuttlebone is an oval, flattened endoskeleton, whose main structural elements are septa and walls/pillars.^61^ These elements are arranged in a carpark structure that comprise chambers enclosed by septa and internally subdivided by walls/pillars.^62,63^ The crystal units that built up *Sepia* cuttlebone are encased by biopolymer membranes (Figure S1, Supporting Information), which can be as thick as 500 nm, and occlude fine, foam-like networks of fibrils.^61^ Samples of both skeletons were collected from the Cantabrian Sea (North Spain) (Figure 1). X-ray fluorescence (XRF) (Bruker S2 Ranger) shows that both mineral components BIO-CAL and BIO-CAL are almost pure CaCO_3_, with minor amounts of Mg and Sr (0.12 wt % Mg and 0.17 wt % Sr in BIO-CAL and 0.13 wt % Mg and 0.17 wt % Sr in BIO-ARG). Each sample was ground using an agate mortar and sieved to separate the selected grain size fraction 125 < Ø < 200 μm. Powdered samples were then cleaned by immersing them in technical grade ethanol (94% isopropanol) in an ultrasonic bath during 10 min. This procedure was repeated three times. Afterward, samples were washed with high-purity deionized water (ρ > 18 MΩ·cm) and then dried for 12 h in an oven at 105 °C.

The specific surface area of the biocarbonate samples was determined by measuring the N_2_ adsorption isotherms. These measurements were conducted at −196 °C in an automatic apparatus (Micrometrics ASAP 2020). Prior to the adsorption measurements, the samples were outgassed in situ under vacuum overnight at 90 °C. It is controversial that this type of measurement is adequate to estimate specific surface areas in systems where organics are present.^64^ In the particular case of biocarbonates, different authors have pointed out that N_2_ adsorption isotherm measurements are likely to be influenced by specific biocarbonate microarchitectures and lead to overestimated reactive surface areas.^65−68^ This inference has been proved for being wrong for biocarbonates like the shells of benthonic foraminifera.^68^ To evaluate the possible contribution of the biopolymers that resulted in an overestimation of the specific surface areas of BIO-CAL and BIO-ARG, N_2_ adsorption isotherm measurements were conducted on both pristine samples and samples thermally treated (2 h at 350 °C in an oxygen atmosphere) to remove most of their biopolymers. The specific surface areas (*S*_BET_) thus determined were 28.59 ± 0.26 and 30.85 ± 0.23 m^2^/g for pristine and thermally treated BIO-CAL, respectively. In the case of pristine and thermally treated BIO-ARG, the determined *S*_BET_ values were 27.49 ± 0.13 and 29.58 0.18 m^2^/g, respectively. The similarity of the *S*_BET_ of the pristine samples and the thermally treated ones indicates that the presence of the polymeric component does not lead to an overestimation of the specific area of the pristine samples. The slightly higher surface area of the thermally treated samples can be explained by the generation of porosity during biopolymer degradation.^69^ Part of this porosity can also be destroyed during the thermal treatment due to recrystallization and abutting of the crystal units. The average biopolymer contents of BIO-ARG and BIO-CAL samples, as determined by thermogravimetric analysis (TGA) in a Mettler Toledo TGA/SDTA 851 thermal analyzer in an oxygen atmosphere, are 9.8 and 1.7 wt %, respectively. These values are in good agreement with previously reported organic contents for *S. officinalis* cuttlebone (9.8 wt %^62^) and scallop shells (1.3 wt %^70^).

### Batch Experiments

Interaction experiments were carried out at 23 °C and atmospheric pressure by placing 200 mg of each powdered pristine hard tissue (BIO-CAL or BIO-ARG) into beakers containing 100 mL of Pb-bearing solution ([Pb]_aq_= 10 mM) and a floating magnet. The Pb-bearing aqueous solution was prepared by dissolving reagent grade Pb(NO_3_)_2_ (Sigma-Aldrich) in ultrapure deionized water. Borosilicate glassware (VWR) was used to perform all of the experiments. Beakers were sealed (*V*_total_ = 150 mL) with Parafilm to avoid water evaporation during experiments. A suspension of skeleton fragments in Pb-bearing solution was stirred at 300 rpm with a multiposition magnetic stirring plate during the entire duration of experiments lasting for 4 h and 1, 2, 3, 5, 7, and 10 days. Independent experimental runs were conducted for both biogenic materials. Experimental runs were duplicated to confirm the experimental reliability and to determine the standard deviations. After the end of each experiment, the solution was filtered under low vacuum using 0.45 μm Nitrocellulose filters (Millipore, Ø = 0.45 μm). Recovered solids were then dried at room temperature and stored in plastic Petri dishes containing a filter for gravimetric analysis to decrease the relative humidity. This experimental procedure has been previously used by the authors to study Pb sorption by purely inorganic CaCO_3_ (calcite and aragonite). Therefore, the kinetic data obtained in the current study allow for a fully consistent comparison with those derived from our previous work.^29^

### Analytical Methods

The mineralogy of both pristine and reacted skeletons was characterized by X-ray powder diffraction (XRD) using a PANalytical Xpert Pro equipped with a Cu X-ray source (working at 40 kV and 40 mA) and a zero silicon holder. X-ray patterns were recorded between 5° and 70° 2θ, with a step range of 0.017° and a measured time per step of 80 s. XRD patterns were used to identify and semiquantify (Rietveld refinements) the phases with X’Pert HighScore Plus (PANalytical B.V., Erie, PA, USA) software. The diffraction patterns were compared to standard mineral files for calcite (PDF 05-0586), aragonite (PDF 41-1475), cerussite (PDF 47-1734), and hydrocerussite (PDF 13-0131). Reacted samples were further analyzed by scanning electron microscopy (SEM). Backscattered electron (BSE) and secondary electron (SE) images were taken on polished gold-coated cross sections of epoxy-embedded samples using a JEOL-6610LV microscope equipped with energy-dispersive X-ray spectroscopy (EDX, INCA Energy 350). The pH of the initial Pb-bearing solutions (4.3 ± 0.05) was measured using a Thermo Scientific (Tokyo, Japan) Orion Versa Star Pro system. Finally, the total concentration of lead (Pb) and calcium (Ca) in the liquid samples was analyzed by inductively coupled plasma optical emission spectrometry (ICP-OES) (Agilent Varian, 700 ES).

### In Situ AFM Observations

The interaction between millimeter-sized fragments (∼2 × 2 × 2 mm) of *S. officinalis* and 10 mM Pb-bearing aqueous solutions was studied at the nanoscale with a Cypher ES atomic force microscope. The images were recorded in tapping mode at 25 °C using ultrahigh-frequency tips (NanoWorld Arrow UHF-AuD). The topographies of the mineral surface were acquired with a line-scanning rate of 1.8 Hz during and after the injection of the solutions. The only mathematical treatment applied to the images was a zero-order flattening executed by the default software of the Cypher Asylum. Individual representative samples were carefully prepared for the experiments by cutting the initial bigger specimens with a stainless steel blade. Samples were stuck on a magnetic holder (Ted Pella) using an adhesive for microscopy, Leit-C. The samples were initially observed in ultrapure water for 2 h. After locating a suitable surface with optimal scanning parameters, the Pb-bearing solution was injected into the AFM cell and the interaction process was observed continuously up to 7 h. To confirm the reproducibility of the observations, different locations on the same crystal have been observed before concluding each experiment. The entire experimental procedure has been replicated two times. Profile measurements were made with the open source software Gwyddion 2.59.^71^

## Results

### Biomaterials Interacting with a Pb-Bearing Aqueous Solution

XRD confirmed that the only mineral components present in the pristine samples of the *C. opercularis* (*A. opercularis*) shell (BIO-CAL) and *S. officinalis* cuttlebone (BIO-ARG) are calcite and aragonite, respectively. The interaction between Pb-bearing aqueous solutions and grains of biogenic materials (BIO-CAL and BIO-ARG) was studied by characterizing mineralogical changes in the reacted samples compared to the pristine ones as well as chemical changes in the liquid phase as the reaction proceeds in batch experiments.

**Figure 2:**
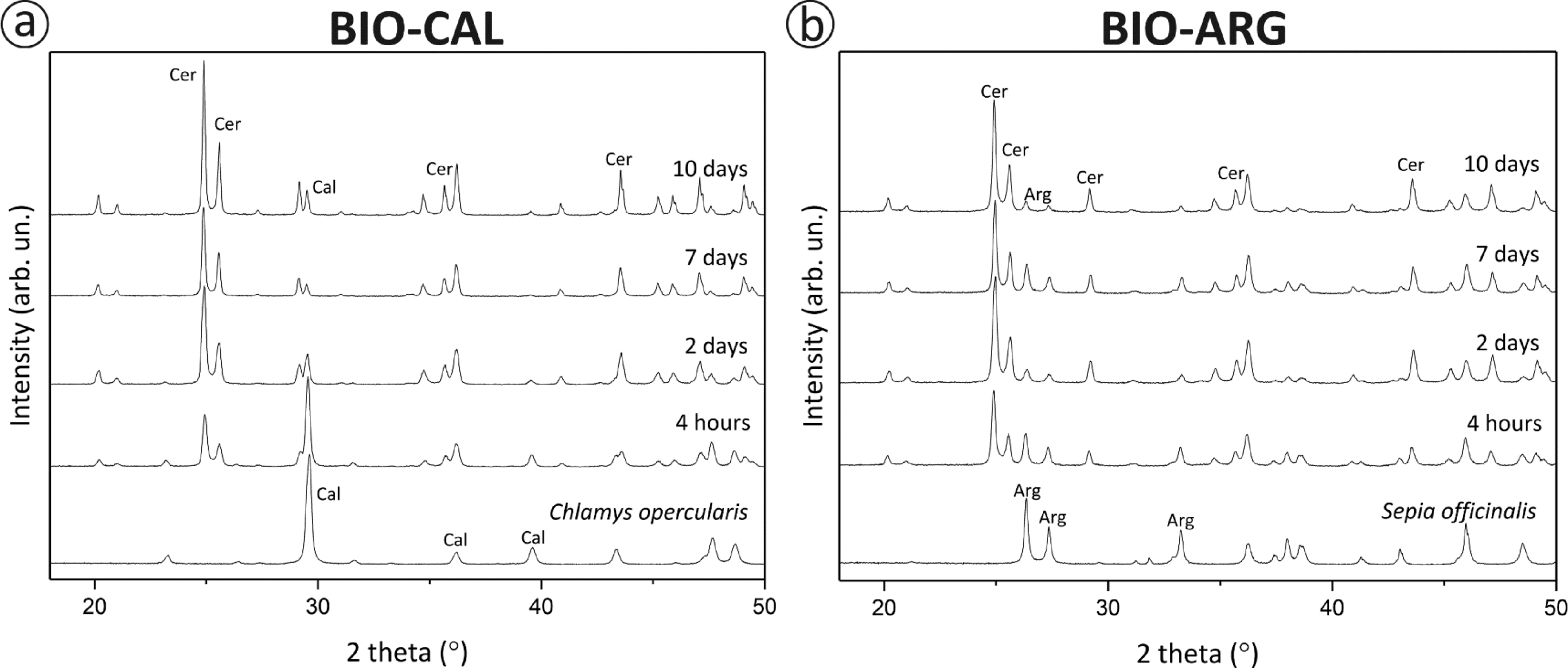
X-ray diffraction patterns of *C. opercularis* (*A. opercularis*) (BIO-CAL) (a) and *S. officinalis* (BIO-ARG) crystals (b) after different times of interaction with a 10 mM Pb-bearing aqueous solution.

The X-ray diffraction patterns shown in Figure 2 correspond to BIO-CAL and BIO-ARG samples after interaction times in contact with Pb-bearing aqueous solution. In addition to the characteristic diffraction peaks of calcite (BIO-CAL) and aragonite (BIO-ARG), the diffractograms of both biogenic materials (Figure 2a,b) show new slightly broad peaks at 2θ = 24.90° and 25.59° shortly after the beginning of the interaction (4 h). These new peaks can be attributed to cerussite. With the interaction time, an increase in the relative intensities of the cerussite peaks was also observed. The peak position remained unchanged during the experiment, although the cerussite peaks became narrower with time. The results of XRD Rietveld analysis indicate that after 4 h contact time with the Pb-bearing solution, BIO-CAL and BIO-ARG samples contain 5.3 and 6.0 mol % cerussite, respectively. After 3 days, the cerussite content goes up to 23.7 mol % in reacted BIO-CAL and 34.2 mol % in reacted BIO-ARG. Finally, after 10 days of interaction, reacted BIO-CAL and BIO-ARG samples contain 44.2 and 32.6 mol % cerussite, respectively. The results indicate fast initial precipitation of cerussite in the presence of BIO-ARG, followed by a stagnation of reaction rate. On the contrary, steady cerussite precipitation leading to a larger reaction yield after long interaction times is observed in experiments with BIO-CAL samples.

**Figure 3:**
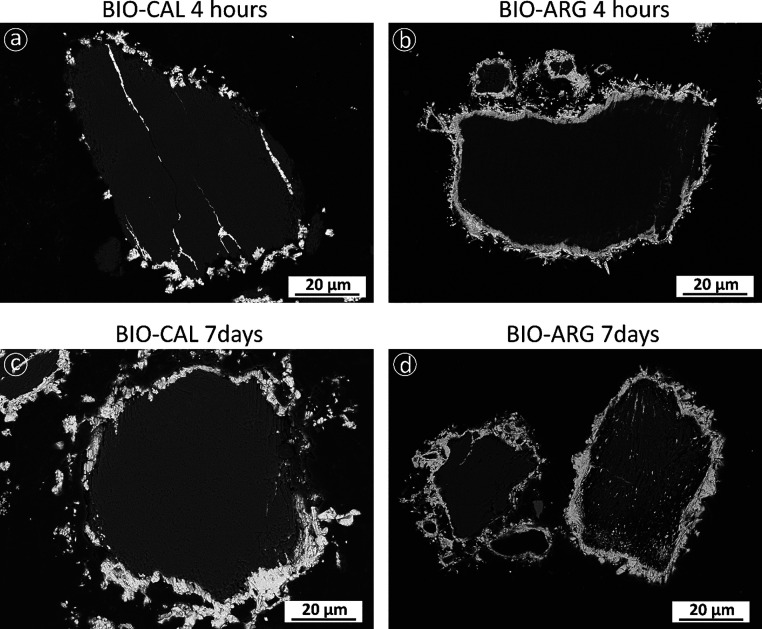
SEM micrographs of the cross section of BIO-CAL and BIO-ARG samples reacted with 10 mM Pb(NO_3_)_2_ aqueous solution at different interaction times.

**Figure 4:**
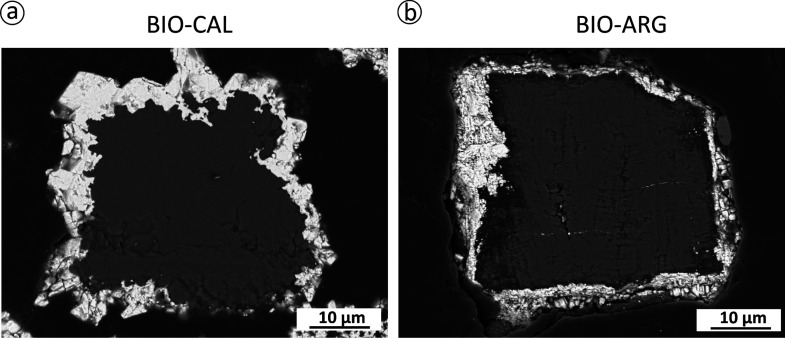
BSE-SEM micrographs of cross sections of reacted crystals after 7 days of interaction with Pb-bearing aqueous solutions: (a) The BIO-CAL core is rounded by an incomplete layer of cerussite crystals showing euhedral morphology. (b) The BIO-ARG surface is completely covered by cerussite crystals showing sizes lower than those observed in BIO-CAL.

Backscattered electron microscopy imaging of cross-cut sections of reacted BIO-CAL and BIO-ARG samples shows that these samples consist of a large dark core surrounded by a bright rim (Figure 3). The transition between the rim and the core is sharp, and no significant gap is observed at the rim–core interface. The EDX analysis of cores and rims in both types of samples yields highly homogeneous compositions that are consistent with the cores composed of a CaCO_3_ polymorph and the bright rims consisting of cerussite. In the case of reacted BIO-CAL samples, cerussite rims are initially thin (thickness, ∼3 ± 0.9 μm; interaction time, 4 h) and very patchy but become thicker (thickness, ∼8 ± 2 μm; interaction time, 7 days) and less patchy as the interaction progresses (Figures 3a–c). Moreover, cerussite crystals in the rim can reach sizes as large as 10 μm and develop euhedral morphology after 7 days of interaction (see, for example, crystals at the top left corner in Figure 4a). However, a continuous cerussite layer around the calcite core fails to form, leaving small domains of the surface of BIO-CAL sample grains uncovered by cerussite, even after long interaction times (Figures 3c and 4a). In contrast, cerussite rims formed around aragonite cores in reacted BIO-ARG appear as continuous layers that resemble the original shape of the pristine samples after short interaction times (Figure 3b). An increase in the rim thickness with the interaction time is also observed in this case. Rims are ∼5 ± 0.9 and ∼8 ± 0.6 μm thick after 4 h and 7 days of interaction, respectively (Figure 3b–d). Rims in BIO-ARG samples consist of smaller cerussite crystals compared with the ones observed in reacted BIO-CAL sample rims (Figure 4b).

**Figure 5:**
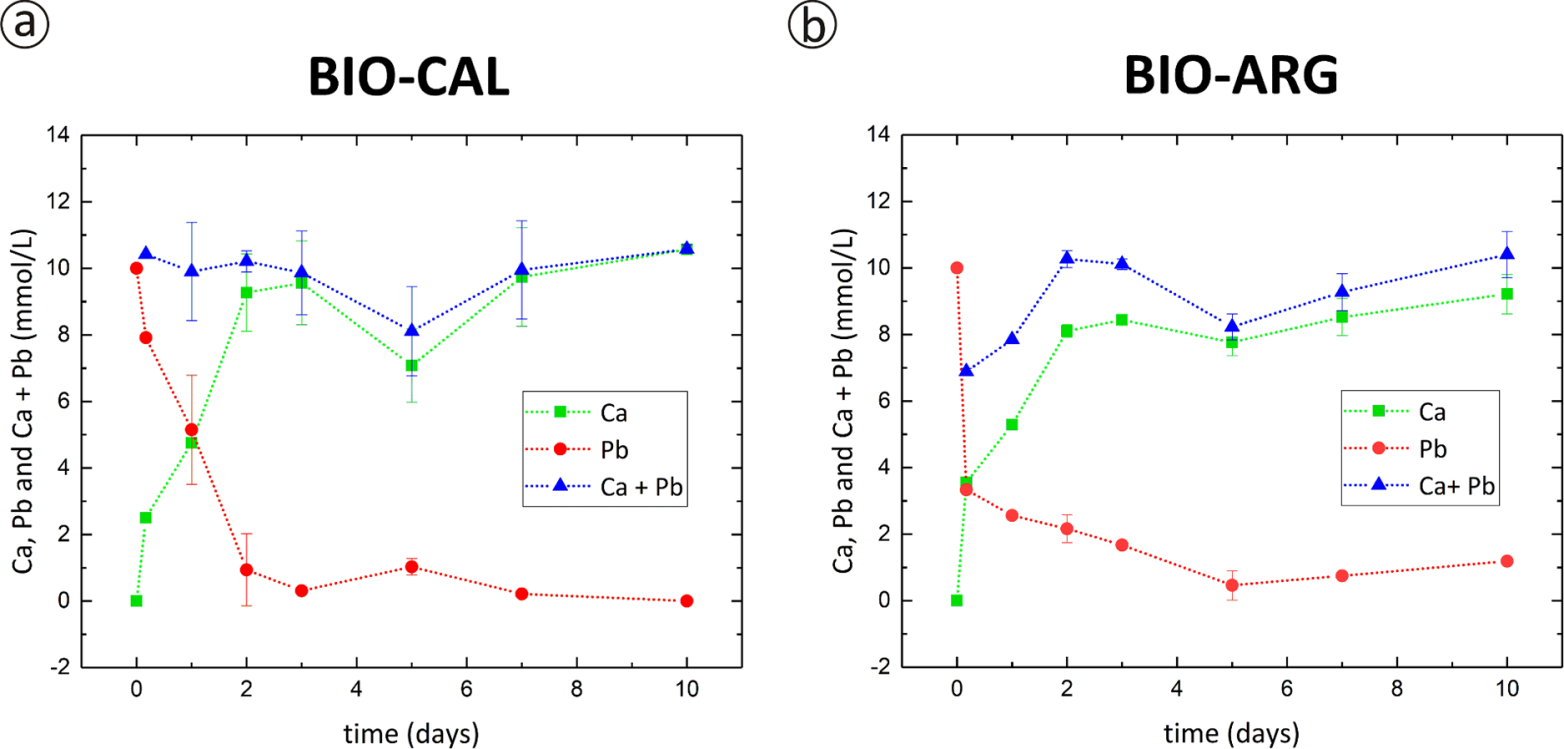
Evolution of Pb and Ca concentrations as a function of time for interaction experiments carried out with [Pb]_*i*_ = 10 mM obtained by ICP-OES: (a) *C. opercularis* (*A. opercularis*) (BIO-CAL); (b) *S. officinalis* (BIO-ARG).

The compositional evolution of the aqueous phase during the interaction between the Pb-bearing aqueous solutions and the BIO-CAL or BIO-ARG samples is monitored by ICP-OES analysis. Plots of Ca and Pb concentrations against interaction time are shown in Figure 5. As can be seen, the Pb concentration in the aqueous solution undergoes an early rapid drop (during the first 4 h) followed by a slower decrease, regardless of the material used in the interaction experiments. The total Ca and Pb concentrations remain nearly unchanged. Thus, the observed decrease in the Pb concentration is reciprocally correlated with an increase in the Ca concentration. A closer inspection of the data plotted in Figure 5a,b reveals some differences in the removal rate of Pb from the liquid phase depending on the type of biogenic material used in the experiments. In experiments conducted using BIO-ARG samples, the Pb concentration drops very fast during the first 4 h and reaches ∼4 mmol/L. At longer interaction time, the rate of Pb precipitation slows down. The Pb concentration reaches values around 2 mmol/L after 48 h and approximately 1 mmol/L at the end of the experiment after 10 days (Figure 5b). In experiments conducted with BIO-CAL samples, the Pb concentration drops at a significantly slower rate. After 4 h of interaction, the Pb concentration in solution reaches a value of ∼8 mmol/L. Afterward, the rate of Pb concentration decreases slows down but less strongly compared to the corresponding experiments with BIO-ARG. Pb reaches a value of ∼1 mmol/L after 48 h and is ∼3 μmol/L after 10 days (Figure 5a). Therefore, during the first 4 h of interaction, the BIO-ARG surface appears as a more efficient Pb scavenger, which takes up ∼79% of the initially dissolved Pb, whereas BIO-CAL takes up only ∼50% in the same interaction period. The Pb uptake efficiency is inverted at longer interaction times. After 10 days of interaction, ∼99.9% of dissolved Pb has been scavenged from the solution by BIO-CAL and only ∼90% by BIO-ARG. The observed differences in the amounts of Pb removed from the solution, estimated by the ICP analysis of the aqueous solution, correlate with the thickness of cerussite layers formed around the grains of carbonates. Longer interaction times result in thicker cerussite rims.

### Surface Reaction with AFM

**Figure 6:**
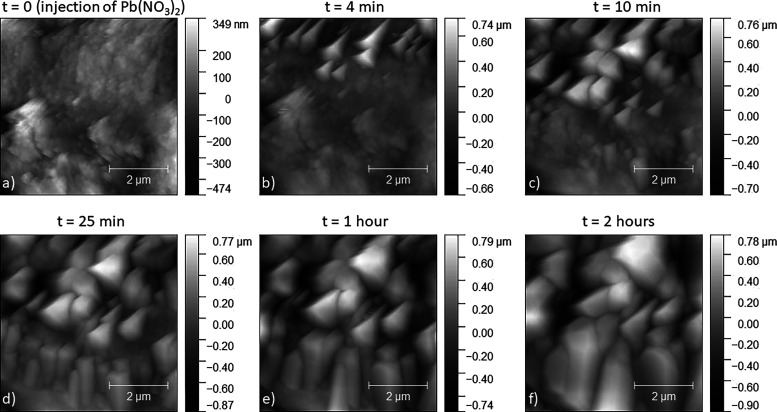
AFM images showing the evolution of the cuttlebone surface after injection of 10 mM Pb(NO_3_)_2_ at different reaction times.

**Figure 7:**
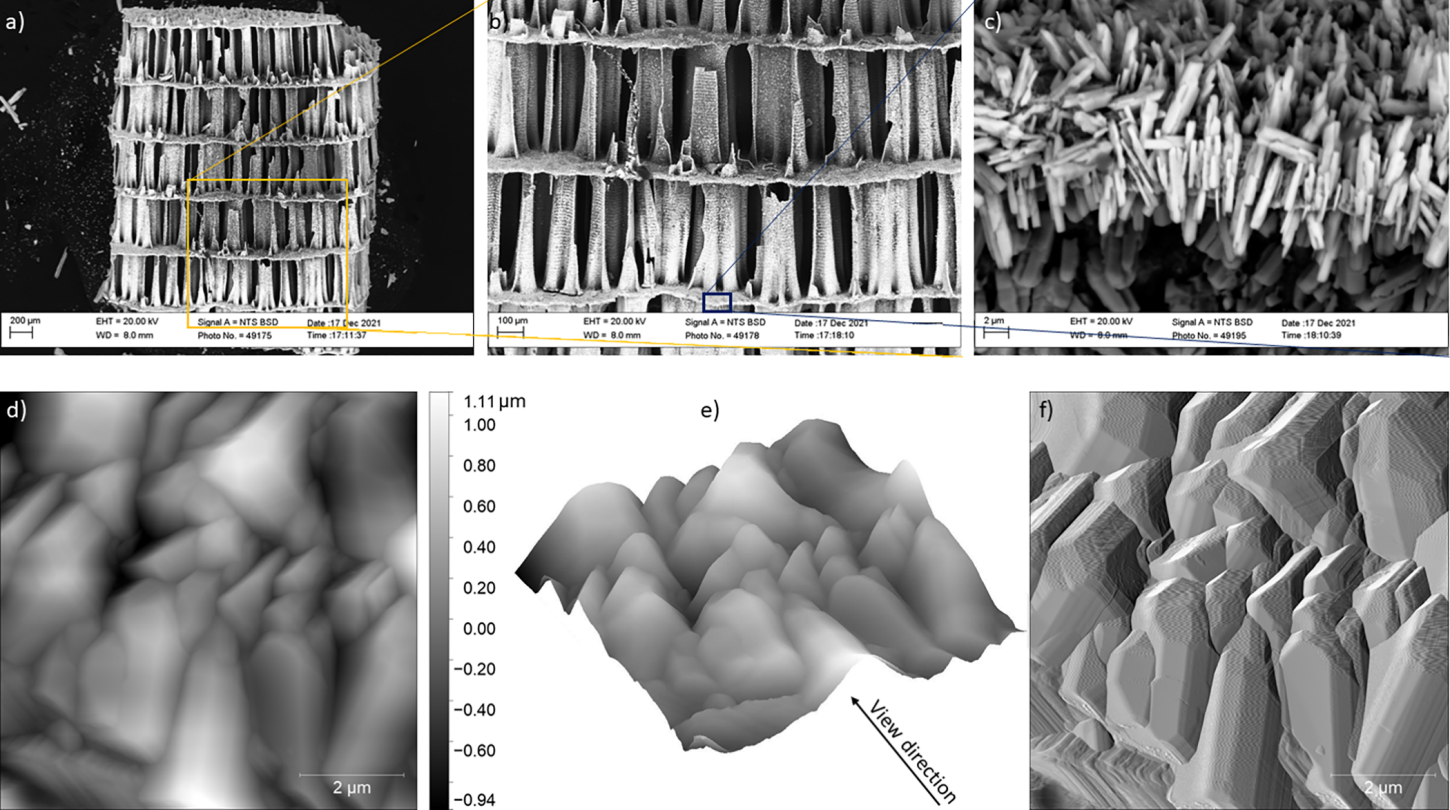
Characterization of the BIO-ARG surface at nanoscales. (a–c) BSE-SEM micrographs recorded ex situ at the end of AFM flow-through experiments (*t* = 7 h): (a, b) the pristine biomineral structure is preserved and (c) the newly formed crystals appear as elongated prisms that grow near perpendicular to the platform surface. AFM images of height channel (d, e) and amplitude channel (f) show that the cerussite crystals grow coaligned on *S. officinalis* surfaces.

The interaction between BIO-ARG surfaces and the Pb-bearing aqueous solution leading to the precipitation of PbCO_3_ was in situ-monitored by AFM. In Figure 6a–f, a sequence of height-channel images shows the evolution of the *S. officinalis* surface in contact with Pb aqueous solution at different reaction times. Figure 6a shows the typical nanogranular appearance of the *S. officinalis* cuttlebone surface prior to the beginning of the interaction. As soon as the Pb-bearing solution is injected into the AFM cell, the formation of nuclei of a new phase on the biomineral surface is observed (Figure 6b). As the Pb-bearing solution–biomineral surface interaction progresses, the newly formed nuclei rapidly grow and coalesce (Figure 6c) to form a layer that completely carpets the biomineral surface after interaction times as short as 25 min (Figure 6d). After 2 h of interaction, newly formed crystals in this layer exceed 2 μm and appear to be strongly coaligned (Figure 6d). SEM images in Figures 7a–c show a*S. officinalis* cuttlebone sample after 7 h of interaction with the Pb-bearing solution. It is worth noting that the carpark structure characteristic of the pristine biomaterial, with evenly spaced platforms interconnected by pillars, is preserved throughout the interaction (Figure 7a,b). Inspection of this structure under higher magnification shows that elongated prismatic crystals of newly formed crystals reacted with the*S. officinalis* cuttlebone. These crystals appear arranged with the prism length approximately perpendicular to platform surfaces in the cuttlebone carpark structure (Figure 7c). Cerussite crystals formed on the surface of cuttlebone pillars show features and orientations identical to those in the platforms. In situ AFM imaging of the surface of septa and pillars of reacted cuttlebone (reaction time, 7h) confirms that crystals of the new phase grow aligned to each other (Figure 7d–f).

## Discussion

### Removal of Dissolved Pb by Biogenic Calcium Carbonates: Efficiency and Mechanisms

**Figure 8:**
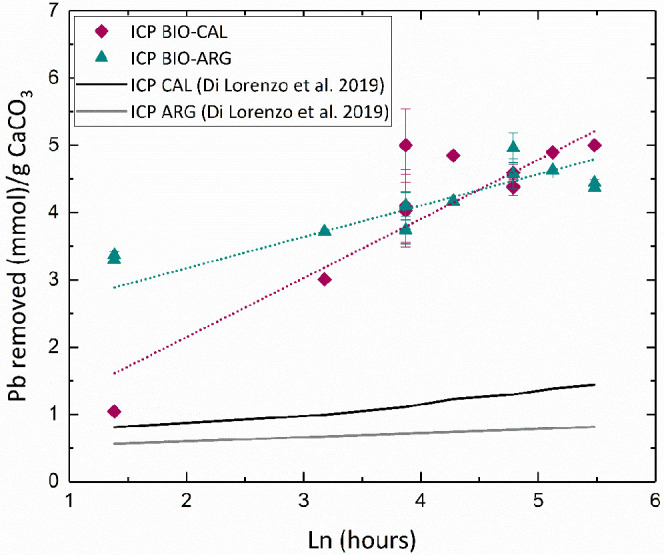
Plot of Pb uptake by BIO-CAL and BIO-ARG against interaction time (in logarithmic units). Data of Pb uptake by geological calcite and aragonite^29^ are also plotted for comparison. Note that Pb uptake data are mass-normalized.

Figure 8 illustrates the variation of Pb taken up as a function of time during the interaction of a solution containing 10 mM Pb with (i) fragments of the biogenic carbonates of *C. opercularis* (*A. opercularis*) shell (BIO-CAL) (pink rhombus) and *S. officinalis* cuttlebone (BIO-ARG) (blue triangles) (this work) and (ii) equally sized fragments of abiogenic calcite and aragonite crystals (lines black and grey).^29^ As can be seen, both biogenic carbonates remove Pb from the aqueous phase at a much faster rate than that of their geological counterparts. The total amount of Pb removed at termination time is overwhelmingly higher when experiments are conducted using biomineral fragments, resulting in the removal of up to 99.9 and 90.0 mol % of the initial dissolved lead by BIO-CAL and BIO-ARG, respectively. Only Godelitsas et al.^21^ have reported similarly high Pb removal efficiency in the experiments conducted with calcium carbonate mineral of geological origin whose grain size and surface area were similar to those of the samples in this study. However, it is worth mentioning that in the experiments conducted by Godelitsas et al.,^21^ the initial concentration of dissolved Pb was significantly lower (5 mM) and the solid/liquid ratio (0.01g/mL) was significantly higher than in the experiments in this study, which were conducted using the exact same conditions as Di Lorenzo et al.^29^

Three main mechanisms can be responsible for the removal of heavy metals from aqueous solutions by mineral surfaces: (i) formation of chemical bonds that can be strong (chemisorption) or weak (nonspecific adsorption), (ii) solid solution formation through solid-state in- and out-diffusion ions in the near surface region, and (iii) through precipitation of secondary minerals.^72−74^ Pb sorption mechanisms at the abiogenic calcite or aragonite–aqueous solution interface have extensively been studied combining multiscale approaches.^19−25,28−31^ There is a consensus that the interaction of Pb-bearing aqueous solutions with geological calcite or aragonite Pb leads to the surface precipitation of lead carbonates (cerussite and hydrocerussite). Potential Pb absorption into the calcite or aragonite crystal lattice is negligible due to the extremely low solid-state diffusion rate under ambient condition, which is confirmed with transmission electron microscopy observation of the interfaces.^34,75^ Moreover, spectroscopic analyses have confirmed that surface adsorption of Pb onto calcite and aragonite crystals is only relevant at the very early stages of the mineral–fluid interaction process or in a very diluted solution.^21^ The larger surface area of calcium carbonate biogenic carbonates compared with their inorganic counterparts may be one of the factors that explain the higher uptake of Pb by the former. However, the chemical analyses of the fluid during its interaction with BIO-CAL and BIO-ARG samples show that the decreases in the Pb concentration are coupled to the increase in Ca concentration. This observation indicates that Pb precipitation is coupled with the dissolution of the biogenic calcium carbonate as previously reported in the studies with inorganic calcium carbonate phases.^21^ This interpretation is in good agreement with SEM observations showing the formation of new crystalline phases growing on the surface of BIO-CAL and BIO-ARG samples soon after the beginning of the interaction (Figures 3 and 4). EDX analyses of these crystals showed that they contain Pb. However, the amount of removed Pb estimated from the chemical analysis of the aqueous solution (Figure 8) can include a contribution that does not correspond to cerussite precipitation but to Pb adsorption by biopolymers exposed on biocarbonate surfaces. Furthermore, XRD analysis of interacted samples confirms that they consist of a mixture of the initial calcium carbonate phase and secondary cerussite (Figure 2). The Pb removal estimated from XRD analyses of interacted biominerals confirms higher effectiveness of both biominerals (3.25 mmol cerussite/g BIO-ARG and 4.68 mmol cerussite/g BIO-CAL) than those previously reported for geocarbonates (1.33 mmol cerussite/g aragonite and 1.58 mmol cerussite/g calcite).^29^ Future works will investigate the contribution of biopolymers to the Pb removal.

### Reaction Paths and Physicochemical Evolution of the System

Experimental results show that the interaction of a Pb-bearing aqueous solution with BIO-CAL and BIO-ARG leads to surface precipitation of cerussite crystals. At the same time, dissolution of biogenic carbonates Ca^2+^ and CO_3_^2–^ ions is released to the fluid. The reaction between the released CO_3_^2–^ ions and the dissolved Pb^2+^ ions may result in the formation of cerussite crystals as soon as supersaturation for the formation of this phase is attained. This dissolution–precipitation reaction can be described as
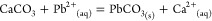
1

The equilibrium constants for aragonite and calcite in reaction 1 are slightly different:
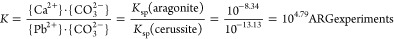
2
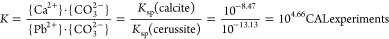
3

The terms in parentheses represent the activities of the aqueous ions. The equilibrium constants for aragonite (*K*_sp_ = 10^–8.34^), calcite (*K*_sp_ = 10^–8.47^), and cerussite (*K*_sp_ = 10^–13.13^) have been taken from the llnl.dat database, included in the geochemical code PHREEQC.^76^ Aragonite is slightly more soluble than calcite, and both calcite and aragonite are more than 4 orders of magnitude more soluble than cerussite.

The large solubility difference between both calcium carbonate polymorphs and cerussite explains that as soon as the former phases start to dissolve, the fluid becomes supersaturated with respect to cerussite. Considering the CaCO_3_–PbCO_3_ system as a mechanical mixture of pure end-members,^34^ the equilibrium constant of reaction 1 can be defined as the ratio between the solubility product of each involved calcium carbonate phase and that of cerussite, as described by expressions 2 and 3. It is important to note that the equilibrium constants for aragonite and calcite have been defined without taking into consideration whether these phases have an inorganic or biogenic origin. As explained in Introduction, biocarbonates are composite materials that consist of two intimately interlinked phases, biopolymers and calcium carbonate minerals with mesocrystalline architectures.^53^ In both calcite and aragonite biomaterials, networks of biopolymer fibrils are occluded within the mineral component. Different authors have studied the effect of these intracrystalline organic molecules on the structure of biocalcites, bioaragonites, and biomimetic calcium carbonates.^77−80^ Pokroy et al.^78^ concluded that this occlusion induces anisotropic lattice distortions in biocarbonates of the mollusk phylum. These authors found that intracrystalline organic inclusions cause an increase in the *a*- and *c*-lattice parameters and a decrease in the *b*-lattice parameter of bioaragonite, while they promote the increase in both the *a*- and *c*-lattice parameters in biocalcite. Similar lattice distortions have been observed in crystals formed in the presence of organic macromolecules and biopolymers extracted from calcium carbonate hard tissue shells.^81−85^ Seknazi and Pokroy^86^ have established that high lattice strain in biocarbonates arises from the structural mismatch at interfaces between biopolymers and mineral phases. The existence of a biopolymer occlusion-related lattice strain increases the free energy of biogenic calcium carbonates compared to that of their abiogenic counterparts. Consequently, their solubility is also increased.^69,87−90^ Biocarbonates commonly incorporate small amounts of ionic impurities. As explained in Introduction, both BIO-CAL and BIO-ARG contain minor amounts of Mg and Sr, most likely as isomorphic substitutions in the lattice of their mineral component. These deviations from the composition of each CaCO_3_ counterpart end-member further contribute to the increase in BIO-CAL and BIO-ARG solubility.^88−92^ Moreover, biocarbonates are nanoparticulate, polycrystalline materials. It is well known that crystal size influences solubility and smaller crystals are less stable than larger ones.^93−96^ All these factors result in a larger negative Gibbs free energy change involved in reaction 1 for BIO-CAL and BIO-ARG, which makes it more likely to proceed further above the limits observed for purely inorganic materials.

### Lead Scavenging Capacity of Calcium Carbonate Biomaterials: BIO-Carbonate versus GEO-Carbonates

The higher solubilities of biocalcite and bioaragonite compared to their abiogenic counterparts may also contribute, to some extent, to their enhanced Pb-scavenging capacity. Since a larger amount of biogenic calcium carbonate can be dissolved, the fluid becomes more supersaturated with respect to cerussite and, consequently, a larger amount of this phase will precipitate. As soon as the dissolution–precipitation feedback loop is established, the process will progress, leading to the precipitation of larger amounts of cerussite as long as the loop continues to operate.

It is worth noting that the biopolymeric component of biomaterials comprises both water-soluble and -insoluble macromolecules.^97−99^ During the interaction of Pb-bearing aqueous solutions with biocalcite and bioaragonite, it can be expected that a small amount of the soluble macromolecules exposed on the surface of the biocarbonate will be released to the fluid phase as the calcium carbonate dissolution–cerusite precipitation reaction progresses. Dissolved macromolecules are complex mixtures that may influence the kinetics of the reaction through their functional groups. However, it was experimentally assessed that organic ligands play a minor role in the dissolution of calcite.^100^ Their effect only becomes appreciable for concentrations of organics in the range of 10^–2^ mol/kg, which results in an increase in calcite dissolution rate of around 2.5 times. In the case of the biocarbonates used in this work, BIO-ARG shows the higher content of biopolymers (9.2 wt %), which mostly consist of chitin.^101^ Assuming the organic matter to be entirely composed of the most abundant component, the chitin (C_8_H_13_O_5_N)_*n*_, and this polysaccharide to completely depolymerize into monomeric units, the concentration of dissolved chitin would be around 0.8 × 10^–4^ mol/kg, negligible compared to the concentration of divalent metals in the aqueous solution at any interaction time ([Pb^2+^] + [Ca^2+^] = 0.01 mol/kg). Since a monomer of chitin only contains one carbonyl group and at least two molecules of chitin will be needed to chelate a dissolved divalent ion, under the most favorable conditions, the maximum amount of aqueous cations that could be complexed would be below 4% of those available. Finally, the nanocrystalline nature of biogenic calcium carbonates grants them significantly larger specific surface areas than shown by their inorganic equivalents. Thus, the specific surface areas of BIO-CAL and BIO-ARG are 28.6 and 27.5 m^2^/g, respectively. These values largely exceed those published for inorganic calcite (4.65 m^2^/g) and aragonite (6.8 m^2^/g) samples within the same size range.^29^ A larger specific surface area can translate into a larger reactive surface, which, in turn, results in a faster dissolution of biocarbonates compared to the geological counterparts. Indeed, the measured dissolution rates of BIO-CAL and BIO-ARG in pure water are 1.33 × 10^–12^ and 1.38 × 10^–12^ (mol cm^–2^ s^–1^), respectively. In equivalent experiments, Di Lorenzo et.^102^ determined dissolution rates for 6.45 × 10^–13^ (mol cm^–2^ s^–1^) for geological calcite and 5.15 × 10^–13^ (mol cm^–2^ s^–1^) for geological aragonite. This means that BIO-CAL and BIO-ARG dissolve around 2 and 2.7 times faster than their geological counterparts, respectively. As a consequence, a coupled faster precipitation of cerussite could be expected. Furthermore, the availability of a larger reactive area also provides more space for cerussite nucleation, facilitating the removal of higher amounts of Pb from the fluid. Moreover, it cannot be discarded that dissolved macromolecules may play a role in promoting cerussite nucleation. The formation of porosity is a common process that occurs during interface-coupled dissolution–crystallization reactions. This porosity can have two origins, the first of which is shared with inorganic samples: (i) porosity generated during interface-coupled dissolution–crystallization reactions to balance negative molar volume and/or solubility changes involved in the reaction and (ii) porosity that results from the dissolution/degradation of biopolymers occluded in the biomineral. The first type of porosity cannot form during the transformation of calcium biocarbonate into cerussite because the molar volume change is positive and large enough that it counterbalances the negative solubility change, regardless of the calcium carbonate polymorph considered. In the time length of the experiments conducted, the second type of porosity can form only through the dissolution of water-soluble biopolymeric components exposed on the surface of the biomineral to interaction with the aqueous solution. Not all biopolymers are soluble and biopolymer degradation is a very slow process at low temperatures.^69,103,104^ Since biocarbonate dissolution and cerussite precipitation are concomitant processes, the impact of this newly formed porosity through biopolymer dissolution on the overall Pb scavenging process will largely be modulated by the characteristics of the precipitated cerussite layer.

### Lead Scavenging Capacity of Biogenic Calcium Carbonates: BIO-CAL versus BIO-ARG

**Figure 9:**
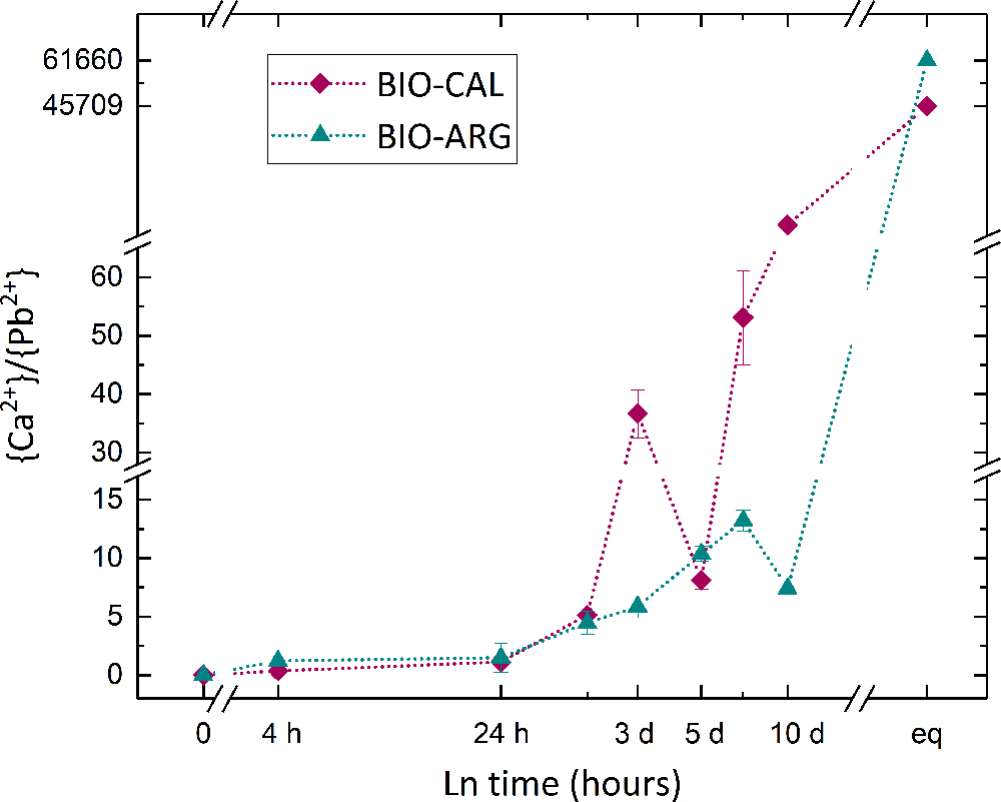
Variation of the Ca^2+^/Pb^2+^ aqueous ion activity ratio in batch experiments.

Despite the close similarity of the thermodynamic driving force controlling the dissolution–crystallization reaction for BIO-CAL and BIO-ARG, the experimental results indicate the differences in the time-dependent reaction yield. Figure 9 shows the time evolution of the cation activity ratio, {Ca^2+^}/{Pb^2+^}, calculated by using the PHREEQC code and the results of ICP-OES analysis of the fluid phase (Figure 5). After 4 h of interaction, {Ca^2+^}/{Pb^2+^} ratios are 0.4 for BIO-CAL and 1.2 for BIO-ARG, reaching after 10 days of interaction maximum values of 3952 and 21 for BIO-CAL and BIO-ARG, respectively. At this point, the system is not yet in equilibrium with the actual phase assemblage, since according to expressions 2 and 3, for this to be the case, the values of ionic activity ratio should be 61660 and 45709, respectively. The precipitated cerussite partially passivates the surface of both calcium carbonate biomaterials, preventing the system from reaching full thermodynamic equilibrium. Thus, only a partial equilibrium is established. In any case, it is worth noting that the maximum values for {Ca^2+^}/{Pb^2+^} ratio observed in the experiments by far exceed the values previously obtained in analogous study with inorganic carbonates ({Ca^2+^}/{Pb^2+^} ∼ 0.5),^29^ which is in agreement with the much higher lead scavenging capacity of biocarbonates.

**Figure 10:**
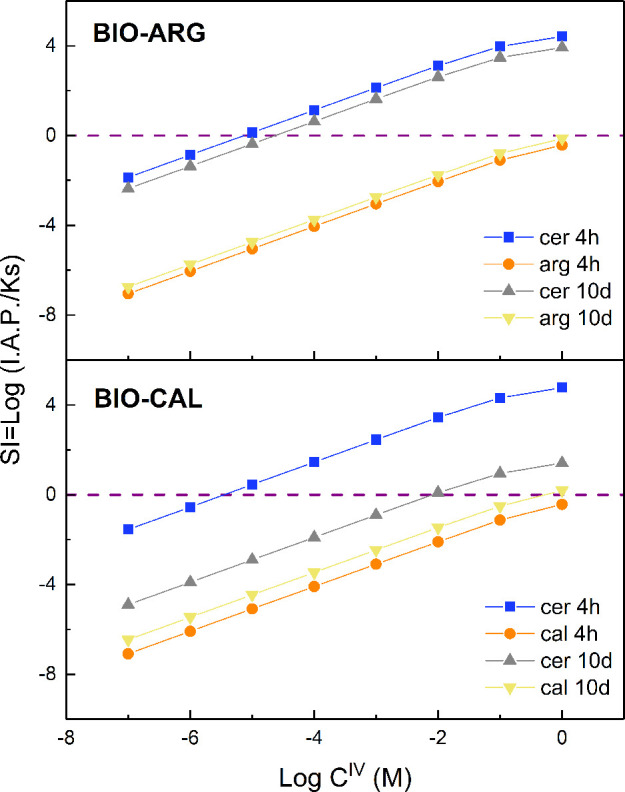
Evolution of the saturation index values of calcite, aragonite, and cerussite during batch experiments (all curves are calculated for pH = 5.4) as a function of total inorganic carbon concentration in log10 units for different interaction times. The equilibrium conditions (SI= 0) are marked by discontinuous lines.

The saturation indexes with respect to the involved calcium carbonate phases are calculated as a function of the amount of inorganic carbon following the expression:
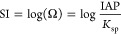
5where IAP is the product of ion activities in the aqueous solution, and *K*_sp_ is the thermodynamic solubility product of the solid phase. Because the acidic pH of the solutions makes it impossible to determine the amount of total dissolved carbon by alkalinity titration, the total carbon was considered as a variable in the description of the evolution of saturation indexes during the interaction process. The saturation in the system is expressed as a function of total carbon considering the range 1 < *C*_tot_ < 10^–7^ M. This broad interval enables a reliable evaluation of the partial equilibrium between the phases involved (Figure 10) as seen in the previous study by Di Lorenzo et al.^29^ Thus, Figure 10 shows the evolution of the saturation indexes with respect to cerussite, calcite, and aragonite. After 4 h, calcite and aragonite are undersaturated for any *C*_tot_ < 1 and, therefore, they are dissolving. The minimum amount of carbon released is 10^–5.6^ M because the formation of cerussite crystals was identified already at this stage by XRD and, therefore, cerussite must be supersaturated. A comparison of the different approach toward the thermodynamic equilibrium depending on BIO-CAL and BIO-ARG, in the period of 4 h to 10 days, is presented in Figure 10. The global equilibrium condition would require that the lines describing cerussite and CaCO_3_ meet at the value corresponding to the equilibrium carbon concentration, which is directly related to the equilibrium pH and partial pressure of CO_2_. In the system with BIO-ARG, the concentration of total carbon that could maintain supersaturation with respect to cerussite is achieved with similar carbonate concentration for 4 h and 10 days. Consequently, the growth of the initially formed cerussite crystals is hindered by the lack of carbon supply from BIO-ARG. The epitactic growth of the product on the surface of the substrate leads BIO-ARG in a condition of partial equilibrium where there is no direct interface between the solvent and the substrate. On the contrary, in the system with BIO-CAL, a significant increase in the carbonate concentration is required to maintain supersaturation with respect to cerussite between 4 h and 10 days (*C*^IV^ = 10^–5.6^ and *C*^IV^ = 10^–2^, respectively) (Figure 10). This demonstrates that under such a condition that a direct contact between the substrate and the solution can be maintained during the progress of the reaction, the formation of cerussite continues even after a significant reduction of the driving force proportional to the distance between cerussite and calcite lines in Figure 10 . Obviously, this simulation considers that the main lead removal mechanism would be the precipitation of lead carbonates, although the contribution of other sorption reactions such as Pb adsorption on biopolymers cannot be ruled out. The study of the role of biopolymers in the dissolution–precipitation reaction loop process is beyond the scope of this work.

Despite the fact that both BIO-CAL and BIO-ARG strongly outperform their geological counterparts, they are not equally effective at scavenging Pb from polluted aqueous solutions. Moreover, their Pb removal capacity differently evolves as the dissolution–crystallization reaction proceeds. As can be seen in Figure 5, BIO-ARG shows a quick initial Pb uptake, but its Pb removal capacity rapidly decays with the interaction time. In contrast, BIO-CAL steadily uptakes Pb up to 48 h of reaction. This Pb uptake capacity slowly declines afterward. BIO-CAL’s Pb removal capacity outperforms that of BIO-ARG for longer interaction times. Figure 8 shows the ICP-OES results normalized for the total moles of cations in solution (*n*_M2+_ = 1 mmol). In this way, the progress of the reaction can follow considering the amount of dissolved CaCO_3_ or the amount of precipitated cerussite. As can be seen, when the total amount of cerussite precipitated during the experiment is considered, BIO-CAL is an overall better Pb scavenging material than BIO-ARG (Figure 8). This conclusion is in good agreement with the results reported by different authors who compared the Pb scavenging performance of calcite and aragonite of geological origin.^21,29,32−34^ All previous studies have concluded that a significantly higher cerussite yield results from the interaction of Pb-bearing solutions with calcite than with aragonite. The main reason underlying this different behavior of the system studies is the structural epitaxy between aragonite and cerussite and the structural mismatch between calcite and cerussite. By combining BSE-SEM and TEM analyses, Di Lorenzo et al.^34^ demonstrated that cerussite grows on geological aragonite showing a strong preferential orientation, with both phases sharing a coherent interface. These authors interpreted that the geological aragonite surface acts as a template that catalyzes the heterogeneous epitactic nucleation of cerussite crystals. The lower energy barrier associated with epitactic nucleation compared to both homogeneous nucleation and growth on a structurally incompatible template surface explains this catalytic effect of the aragonite substrate. A fast formation and growth of numerous oriented cerussite crystals on the surface of BIO-ARG can also explain the high initial Pb uptake capacity of this material. As can be seen in Figure 7c, cerussite crystals appear highly coaligned with their length perpendicularly oriented to *S. officinalis* septa. Moreover, AFM observations also support an oriented growth of cerussite crystals on BIO-ARG surfaces, at least at early stages of the dissolution–crystallization reaction (Figure 6b–f). A recent in-depth study of the *S. officinalis* cuttlebone microstructure using electron backscattered diffraction (EBSD) has shown that in all its structural elements, septa as well as pillars/walls, the *c*-axis of their constituting aragonite crystal subunits is arranged perpendicularly to their surface and rotates with the surface curvature.^61^ Moreover, Griesshaber et al.^61^ also concluded that biopolymer components of *S. officinalis* cuttlebone consist of a mixture of chitin-protein, which is arranged as cholesteric liquid crystals in both the foam-like network occluded within aragonite crystal units and the membranes that envelop these units. Moreover, these authors interpret that the fabric arrangements of the biopolymer guide the organization of the mineral component in both septa and walls/pillars. The observed arrangement of cerussite crystals, with their length perpendicularly oriented to the BIO-ARG surface, consists of cerussite nucleation being epitactic and aragonite crystals in the substrate and cerussite crystals in the overgrowth sharing the orientation of their *c*-axes (Figure S2, Supporting Information). Moreover, a guiding effect of BIO-ARG biopolymers cannot be discarded on the nucleation of cerussite crystals. The later rapid decline of BIO-ARG Pb uptake capacity can be explained as due to the coalescence of cerussite crystals as their epitactic growth progresses, accompanied by competitive growth that further promotes cerussite crystal coorientation, which results in the formation of a continuous porosity-free cerussite layer that carpets the BIO-ARG substrate. This interpretation is in good agreement with SEM observations showing that BIO-ARG cores appear almost completely coated by neo-formed cerussite rims after only 4 h of reaction (Figure 3b). Cerussite rims only undergo slight thickening during the interval between 4 h and 10 days of interaction with the Pb-bearing solution. This is indicative of a highly effective armoring of BIO-ARG substrates by cerussite rims, which prevents further interaction with the Pb-bearing solution and results in a virtually complete stoppage of Pb removal (Figure 5).

Most studies of cerussite precipitation on a geological calcite substrate concur that no epitactic relationships are observed between the two phases and cerussite crystals grow randomly oriented.^28,29,34,35^ Even if a calcite substrate provides a site for cerussite heterogeneous nucleation, thereby reducing the free energy barrier for cerussite nucleation compared to that for nucleation in the bulk, cerussite nucleation will take place at a slower rate on a calcite substrate than on an aragonite one. This explains that geological calcite, as reported by previous studies, as well as BIO-CAL, as shown here, less efficiently removes Pb at an early stage of the replacement reaction than their respective aragonite counterparts do. Indeed, as can be observed in Figure 3, after 4 h interaction with the Pb-bearing solution, few crystals have formed on the BIO-CAL surface, most of which remains available for interaction with the substrate. The percentage of the BIO-CAL reactive surface that remains uncoated by cerussite crystals decreases as the reaction progresses. The lattice mismatch between cerussite and calcite determines that most Pb removal progresses through the growth of the first formed cerussite crystals rather than through the nucleation of new cerussite crystals on the yet uncoated BIO-CAL surface areas. Thus, as can be seen in Figures 3c and 4a, even after 7 days of reaction, although patches consisting of large cerussite crystals coat most of the BIO-CAL surface, these patches do not constitute a continuous layer, leaving areas of the BIO-CAL surface uncoated and available for continuing interaction with the aqueous phase. Moreover, because cerussite patches consist of randomly oriented crystals, they contain a certain volume of intracrystal pores, some of which are open and connect the cerussite-BIO-CAL interface with the bulk solution.^28^

The importance of the existence or absence of epitactic relationships between crystal phases to predict the likelihood of a dissolution–crystallization reaction resulting in large pollutant uptake yields has been highlighted by the results of numerous previous studies.^21,26,27,37,67,105−107^ As a rule, the existence of a good matching between crystal structures facilitates the epitaxial growth of a pollutant bearing a precipitate on the surface of the primary one and results in a fast initial pollutant uptake. However, it also results in a rapid decrease in the primary phase reactive surface and, consequently, a fast drop in its pollutant uptake capacity. Conversely, the absence of epitaxial relationships between the primary phase and the precipitate commonly guarantees a slower decrease in the substrate reactive surface area. Consequently, the dissolution–crystallization reaction can be sustained for a longer period, giving rise to larger precipitate yields and pollutant uptake. It appears that this rule also applies to biocarbonates as the epitactic growth of cerussite on the surface of BIO-ARG strongly limits its long-term efficiency as a Pb scavenger, while the formation of randomly oriented cerussite crystals on the BIO-CAL surface favors its persistent Pb scavenging activity.

## Conclusions

In this work, we have studied the efficiency and mechanism of Pb removal from electrolyte solution via interaction with calcitic and aragonitic biomaterials. The experimental observations suggest the cerussite formation to be the predominant Pb uptake mechanism, which is controlled by dissolution–precipitation reaction on the surface of biocarbonates. This conclusion is in good agreement with those of previously reported studies on Pb sequestration by inorganic calcium carbonate minerals. Comparison of Pb aqueous concentrations measured at the end of experiment reveals that (i) Pb removal yields are around five times larger for biocarbonates than previously found for inorganic carbonates of geological origin and (ii) the calcite biomineral is an overall more efficient Pb sequester (99.9% Pb removal) than the aragonite one (99.0% Pb removal). We attribute the enhanced Pb scavenging capacity of biocarbonates to their specific microstructure that grants them significantly larger specific areas and reactivity compared to their inorganic counterparts. More efficient Pb sequestration by biocalcite compared to bioaragonite is explained by differences in the degree of structural matching between the substrate and the precipitate. While isostructural relationships between cerussite and aragonite facilitate the initial nucleation of cerussite on bioaragonite, the surface quickly becomes passivated. On the contrary, the structural differences between calcite and cerussite prevent extensive passivation of biocalcite and enable a persistent supply of carbonate ions necessary to maintain supersaturation of the solution with respect to the cerussite event at very low Pb concentrations necessary for cerussite precipitation.

The findings reported in this study support the concept that coupled dissolution–crystallization reactions are the most effective metal sequestering mechanism. The precipitation of heavy metal-containing carbonates appears as efficient immobilization in the form of insoluble minerals. The higher specific surface areas and reactivity of biocarbonates make these excellent candidates for being incorporated into strategies for sequestering Pb^2+^ from contaminated waters. Since the canning industry annually produces large volumes of waste biocarbonates, this incorporation can contribute to the circular economy, providing an added value to these materials. Experiments performed in this study define simplified systems. Further research will be needed to gain knowledge on the factors that may modulate the reactivity of biocarbonates and their ability to efficiently sequester dissolved Pb as well as other heavy metals.

## Supporting Information Available

Original SEM images showing the presence of biopolymer membranes in the pristine sepia cuttlebone; cerussite crystals covering the BIO-ARG surface after (PDF)
