# Supplementary material for: Pb Removal Efficiency by Calcium Carbonates: Biogenic versus Abiogenic Materials
Source: Cryst Growth Des. 2023 Dec 5;24(1):79–92. doi: 10.1021/acs.cgd.3c00517 (PMC10767703; doi:10.1021/acs.cgd.3c00517)
Supplement: Supplementary file 1 — cg3c00517_si_001.pdf [file cg3c00517_si_001.pdf]

Supporting information

Pb removal efficiency by calcium carbonates: biogenic versus abiogenic materials

Ana Roza-Llera[1], Fulvio Di Lorenzo[2], Sergey V. Churakov[2], Amalia Jiménez[1]* and Lurdes Fernández-Díaz[3]

[1] Department of Geology. University of Oviedo, 33005 Oviedo, Spain.

[2] Laboratory for Waste Management, Paul Scherrer Institute, Villigen 5232, Switzerland

[3] Department of Mineralogy and Petrology, Complutense University of Madrid, 28040 Madrid, Spain.

*Email: amjimenez@uniovi.es

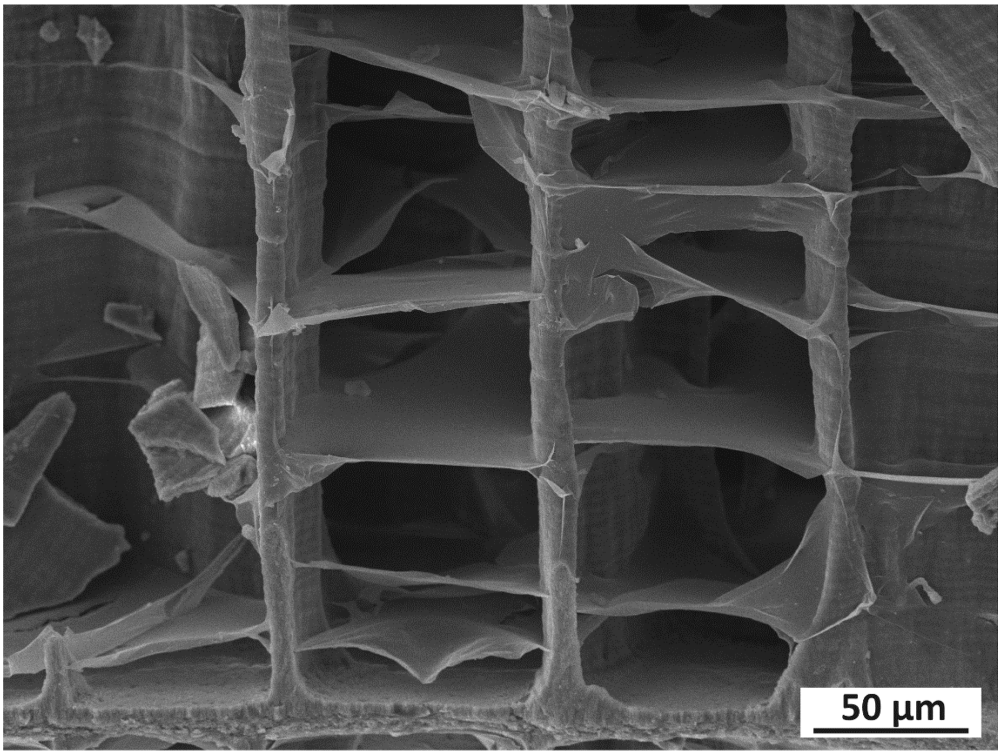

Figure S1: SEM micrograph from a fragment of *Sepia officinalis* cuttlebone showing the biopolymer membranes encasing the crystal units that built up Sepia cuttlebone.

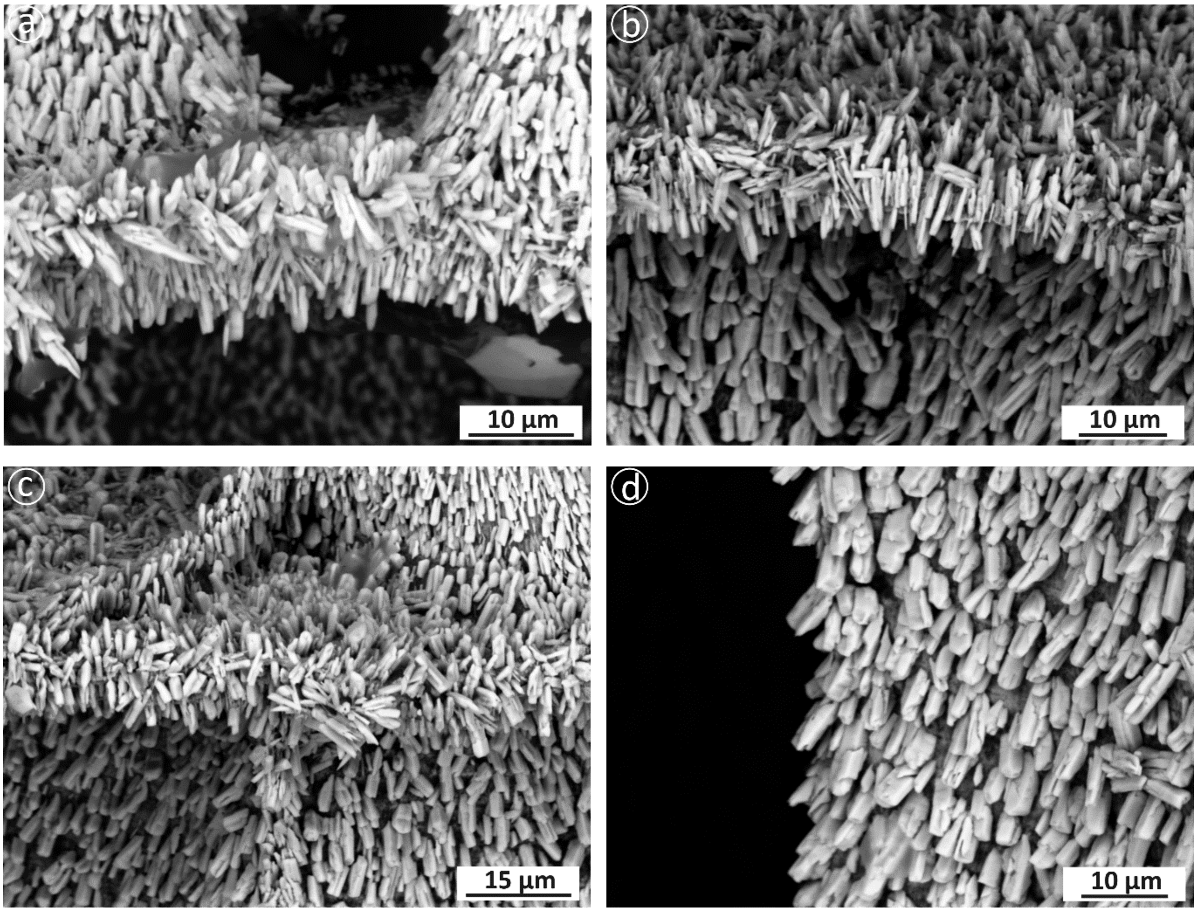

Figure S2: SEM micrographs depicting the cerussite crystals covering the BIO-ARG surface. As can be observed, a high co-orientation of cerussite crystals overgrown on cuttlebone septa (a, b, c) and pillars (d) as well as the approximately perpendicular orientation of these crystals with respect to the BIO-ARG surface.
